# The Association Between Acute Pain and Posttraumatic Stress Symptoms in Children and Adolescents 3 Months After Accidental Injury

**DOI:** 10.1007/s10880-018-9567-6

**Published:** 2018-05-05

**Authors:** Els P. M. van Meijel, Maj R. Gigengack, Eva Verlinden, Alida F. W. van der Steeg, J. Carel Goslings, Frank W. Bloemers, Jan S. K. Luitse, Frits Boer, Martha A. Grootenhuis, Ramón J. L. Lindauer

**Affiliations:** 1 Department of Child and Adolescent Psychiatry, Academic Medical Center, University of Amsterdam, Meibergdreef 5, 1105 AZ Amsterdam, The Netherlands; 2 de Bascule, Academic Center for Child and Adolescent Psychiatry, Amsterdam, The Netherlands; 3 Pediatric Surgical Center of Amsterdam, Emma Children’s Hospital, Academic Medical Center, University of Amsterdam & VU University Medical Center, Amsterdam, The Netherlands; 4 Trauma Unit Department of Surgery, Academic Medical Center, University of Amsterdam, Amsterdam, The Netherlands; 5 Department of Trauma Surgery, VU University Medical Center, Amsterdam, The Netherlands; 6 Emergency Department, Academic Medical Center, University of Amsterdam, Amsterdam, The Netherlands; 7 Pediatric Psychology Department of the Emma Children’s Hospital, Academic Medical Center, University of Amsterdam, Amsterdam, The Netherlands; 8 Princess Maxima Center for Pediatric Oncology, Wilhelmina Children’s Hospital, Utrecht, The Netherlands

**Keywords:** Posttraumatic stress disorder (PTSD), Accidental injury, Pain, Child, Adolescent

## Abstract

Previous research suggests that acute pain is a risk factor for later posttraumatic stress symptoms (PTSS). In a prospective cohort study, we examined the association between acute pain from accidental injury and PTSS in children and adolescents, taking into account factors potentially related to pain or posttraumatic stress. Participants were 135 children and adolescents, 8–18 years old. We measured the worst experienced pain since the accident took place with a visual analogue scale. Three months after the accident, posttraumatic stress was assessed with a self-report measure. We found a positive association between acute pain and posttraumatic stress. The amount of pain was negatively associated with injury severity in girls and positively associated with the presence of an extremity fracture in boys. In children who reported severe pain, this pain was significantly associated with PTSS and may account for around 10% of the variance in the severity of PTSS. Although the experience of pain is subjective, our study indicates that severe pain is associated with the severity of later PTSS. Timely management of pain according to acute pain protocols in all phases and disciplines after accidental injury is therefore recommended.

## Introduction

Every year, many children and adolescents (both groups are referred to as “children” in our study) are injured in accidents and they are often treated in the trauma resuscitation room (trauma room) of the Emergency Department. In a trauma room, a multidisciplinary team of medical specialists and nurses take care of the initial assessment and treatment of trauma patients. Patients are referred to the trauma room in cases with a high-energy trauma mechanism involving a risk of severe and/or potentially life-threatening injuries. A high-energy trauma mechanism refers to mechanisms of injury associated with a high-energy impact such as a fall from height (> 10 ft or 2–3 times the height of the child) or a high-risk automobile crash or a pedestrian/bicycle versus automobile collision (American College of Surgeons, 2012). The accident itself, the injury, the pain, and medical procedures can all be frightening and potentially traumatic (Kahana, Feeny, Youngstrom, & Drotar, 2006; Price, Kassam-Adams, Alderfer, Christofferson, & Kazak, 2016). As a result, children may develop acute stress symptoms. These symptoms disappear spontaneously in the majority of the children in the weeks following traumatic events, but 8–14% develop posttraumatic stress disorder (PTSD) following unintentional injury (Alisic et al., 2014; van Meijel et al., 2015) and up to 18% develop severe posttraumatic stress symptoms (PTSS; Landolt, Vollrath, Timm, Gnehm, & Sennhauser, 2005). According to the DSM-5 classification, PTSD includes symptoms of re-experiencing, avoidance, negative alterations in cognitions and mood and increased arousal, resulting in substantial distress or impairment in functioning (APA, 2013). Acute stress disorder (ASD) can be diagnosed if symptoms persist for no longer than 1 month after the traumatic event; PTSD can be diagnosed if symptoms persist for longer than 1 month (APA, 2013). PTSD is a debilitating psychiatric disorder, often involving the development of co-morbid disorders (Stallard, Salter, & Velleman, 2004) and affecting children’s functioning and physical recovery from injury (Kahana et al., 2006). Since ASD or PTSD at a subsyndromal level can also result in substantial impairment in functioning, it is appropriate to also evaluate and treat children reporting clinically significant persistent PTSS (Gold, Kant, & Kim, 2008).

Most injured patients experience pain, either as a direct consequence of the accident or later on from medical diagnostics and treatment (Baxt, Kassam-Adams, Nance, Vivarelli-O'neill, & Winston, 2004; Keene, Rea, & Aldington, 2011; Melby, McBride, & McAfee, 2011). In a study examining the relationship between acute pain and PTSS in children 8–17 years following traffic-related injury, acute pain was a predictor of PTSS 6 months after the injury, even after controlling for demographic and empirically based risk factors (age, gender, ethnicity, heart rate at triage, prior trauma history, acute stress symptoms, and perceived life threat; Hildenbrand, Marsac, Daly, Chute, & Kassam-Adams, 2016). Pain was identified as a risk factor for ASD in 7- to 18-year-old children after intentional and unintentional injury (Saxe et al., 2005). In turn, ASD is considered to be a risk factor for PTSD (Dalgleish et al., 2008; Saxe et al., 2005). A study of young children with burns identified pain as a factor positively associated with posttraumatic stress outcome (Stoddard et al., 2006). The association between acute pain and later PTSS may be based on shared neurobiological stress mechanisms, enhanced hypothalamic–pituitary–adrenal axis and noradrenergic activation (Asmundson, Coons, Taylor, & Katz, 2002; Hildenbrand et al., 2016; McLean, Clauw, Abelson, & Liberzon, 2005; Norman, Stein, Dimsdale, & Hoyt, 2008). These stress mechanisms, which trigger acute pain, may also serve to encode the memory of the trauma and trigger a posttraumatic stress-related process. Memories of painful events are readily retrievable, indicating that strong encoding occurs at the time the pain was experienced (Morley, 1993; Norman et al., 2008). Pain associated with traumatic injury may act as a reminder of the traumatic event, which may further reinforce memories associated with the traumatic event (Gold et al., 2008). Additionally, the finding that aggressive pharmacological pain management can reduce the likelihood of PTSD lends further support to the relationship between pain and later PTSD development (Gold et al., 2008).

Studies on prediction of, and risk factors for, PTSD generally use clusters of factors and study their combined predictive value or combined risk for PTSD. So far, acute pain has not been included in a screening instrument for risk for PTSD in children following accidental injury (Brosbe, Hoefling, & Faust, 2011; van Meijel et al., 2015; Winston, Kassam-Adams, Garcia-Espana, Ittenbach, & Cnaan, 2003) nor has it been used as stand-alone screener for risk of PTSS. However, the assessment of acute pain is, or easily can be, included in ambulance and emergency care protocols, thus offering an opportunity to identify children at risk for PTSD or PTSS.

Research on the relationship between acute pain and PTSS following child accidental injury is still scarce. If we confirmed or further clarified the above-mentioned initial research findings on the role of acute pain in later child PTSS, we would be able to contribute to screening methods for identifying children at risk and consequently to the prevention of PTSD and PTSS. The aim of this study was to examine the association between acute pain after accidental child injury and PTSS 3 months later, taking into account clinical and demographic factors (gender, presence of an extremity fracture, injury severity, length of hospitalization) potentially related to pain or posttraumatic stress. As the prevalence and the risk of PTSD differs between boys and girls (Alisic et al., 2014; Stallard et al., 2004; Winston et al., 2003), we also examined associations between the variables of interest separately for boys and girls.

## Methods

### Participants and Procedures

For the current study, we used data that were collected as part of the STEPP study in the Netherlands. In the STEPP study, we evaluated a screening instrument (Screening Tool for Early Predictors of PTSD; STEPP) for risk of PTSD in children who had been injured due to accidental trauma (van Meijel et al., 2015). The STEPP study was performed at two academic hospitals in Amsterdam, the Netherlands: Academic Medical Center (AMC) and VU Medical Center (VUmc). Both hospitals are Level 1 trauma centers. The Medical Ethical Committees of both hospitals approved the study. We used the registry systems of the Trauma surgery and Emergency Departments to identify children eligible for this study. We contacted children from 8 to 18 years old (usually via their parents), who had been injured in an accident and were screened for trauma in the trauma room of the emergency department. If children had already been discharged from the hospital, we phoned and asked for an appointment at home. If children were hospitalized, we first consulted the responsible nurse. Participation was only possible after written informed consent was obtained from parents (until the age of 16) and children themselves (from the age of 12). Children were excluded if they had stayed on Intensive Care Units (pediatric or regular) for more than 1 week, or if they were incapable of answering the questions or completing the questionnaires due to cognitive limitations. The first assessment took place after consent was provided. The mean number of days between the accident and the first assessment was 5.8 (SD = 3, range 1–14). The first two authors (EM and MRG) recruited participants, completed informed consent procedures, and collected the data. The sample size for the STEPP study was 161; for the current study, 135 participants with pain data were included in the analysis. Details on recruitment and retention are provided in van Meijel et al. (2015). Demographic and clinical child characteristics are reported in Table 1.

**Table 1:** Sample characteristics and differences between boys and girls

	Total group (*n* = 135)				Girls (*n* = 56, 41.5%)				Boys (*n* = 79, 58.5%)				Difference	Effect size *d*
	*M*	SD	Median	Min–Max	*M*	SD	Median	Min–Max	*M*	SD	Median	Min–Max	*p* Value^a^	
Age^b^	14.0	2.9	15.0	8–18	13.8	2.8	14.0	8–17	14.1	3.0	16.0	8–18	.34^c^	.10
CRIES score	15.2	13.3	12.0	0–61	18.9	15.1	16.5	0–61	12.5	11.3	11.0	0–47	.01^c^	.48
Worst pain	6.8	2.4	7.0	0–10	7.2	2.5	7.3	0–10	6.6	2.3	6.8	2–10	.10^c^	.25
ISS^d^	6.5	7.4	4.0	0–34	5.5	7.0	4.0	0–25	7.3	7.7	5.0	0–34	.06^c^	.24
Days in hospital	3.4	5.5	1.0	0–33	2.7	4.6	0.8	0–22	3.9	6.0	1.0	0–33	.08^c^	.22
Extremity fracture (*n*, %)	38	28			11	20			27	34			.08^e^	.57

*CRIES* Children’s Revised Impact of Event Scale, *ISS* Injury Severity Score, *M* mean, *SD* standard deviation

^a^*p* Value of the difference between boys and girls

^b^Age at the time of posttraumatic stress assessment

^c^Mann–Whitney *U* test was used

^d^ISS score of 1 boy is missing

^e^Fisher’s Exact test was used

### Measures

#### Acute Pain

At the first assessment, we asked children to rate the worst pain since the accident. For this purpose, we used the Visual Analogue Scale (VAS), a small ruler with a 10-cm line, marked with “no pain” on the left, and “the worst possible pain” on the right. The children used a sliding gauge to mark the location corresponding to the amount of pain they had experienced. The reverse side of the instrument shows the corresponding values from 0 to 100 mm. This instrument was used according to internal hospital guidelines (Baas & Kramer, 2008). In the analyses we used the total pain score; a higher score indicates greater pain intensity. Scores can also be rounded to the nearest integer and categorized as *no or mild pain* (0–3), *moderate pain* (4–7), and *severe pain* (8–10). We used these categories to describe the distribution of pain severity in the sample and to examine associations with severity of posttraumatic stress per category.

#### Posttraumatic Stress

Three months after the accident, children completed the Dutch version of the Children’s Revised Impact of Event Scale (CRIES; Children and War Foundation, 1998; Olff, 2005; Verlinden et al., 2014). The Dutch version of the English CRIES was obtained through a standard forward–backward translation procedure by independent health professionals (Verlinden et al., 2014). This self-report measure is based on the definition of PTSD according to DSM-IV-TR criteria and gives a good indication of the presence of PTSD (APA, 2000; Verlinden et al., 2014). It consists of 13 questions in the subscales re-experiencing, avoidance, and hyperarousal, with answers on a 4-point scale. Examples of typical CRIES items are: “Do you startle more easily or feel more nervous than you did before it happened?” and “Do pictures about it pop into your mind?”. We asked the children to focus on their accident when answering the questions. Items are rated according to the frequency of their occurrence during the past week (*Not at all* = 0, *Rarely* = 1, *Sometimes* = 3, and *Often* = 5). The Dutch CRIES is an effective and valid tool for screening of PTSD and shows moderate to good reliability: Cronbach’s alpha for the total score is .89 and for the subscales of re-experiencing, avoidance, and hyperarousal .82, .77, and .74, respectively (Verlinden et al., 2014). In the current study, we used the CRIES total score. The total score can range from 0 to 65, and is an indicator of the child’s perception of posttraumatic stress; a higher total score indicates higher severity. The cut-off score for a positive test is 30. The outcome correlates highly with the PTSD diagnosis according to the Anxiety Disorders Interview Schedule for DSM-IV, Child and Parent Version (ADIS C/P; Verlinden et al., 2014). For the current sample Cronbach’s alpha was .87 (van Meijel et al., 2015).

#### Clinical Information

Information on the presence of an extremity fracture and the length of hospitalization was obtained from the medical records, including the ambulance and Emergency Departments reports. The Injury Severity Score (ISS) was obtained from the trauma registry. In the trauma registry, part of a national trauma registry system, trained data managers register prehospital, in-hospital, and discharge data on injury mechanism, vital signs, type and severity of injuries, treatment, and outcome. The purpose of the national registry system is to be able to evaluate and improve quality of trauma care in the Netherlands. The ISS is a method for describing the severity of injuries in trauma patients. It is related to the likelihood of survival after injury. The ISS is determined by rating the severity of each injury in six body regions (head, neck, face, chest, abdomen, extremity, and external) on the six-point Abbreviated Injury Scale (AIS). The AIS score per body region has a range of 1 (*minor injury*) to 6 (*unsurvivable injury*). The ISS is derived from the sum of the squares of the AIS score of the three most severely injured body regions and has a range of 0–75 (i.e., 5^2^ + 5^2^ + 5^2^). If an injury is assigned an AIS of 6 (*unsurvivable injury*), the ISS score is automatically set to 75. Injury severity can be divided into six categories: *minor* (1–8), *moderate* (9–15), *serious* (16–24), *severe* (25–49), *critical* (50–74), and *maximum* (75) (Baker, O'Neill, Haddon, & Long, 1974; Saxe et al., 2005).

### Statistical Analyses

The data we have presented here originated from a previous study (van Meijel et al., 2015) that aimed to validate the STEPP using responses from 150 participants. This sample size was based on three assumptions: that there would be a prevalence of PTSD of 25%, that the STEPP would have a sensitivity of 90% to identify children at risk for PTSD, and that a 95% confidence interval with limits of 75 and 97% for the sensitivity was required. A total of 161 participants were included. As this paper describes a convenience sample from the earlier STEPP study, no formal power analysis was performed for the current study. However, a post hoc power analysis assuming one sample and a correlation of .3 showed that a sample of 135 patients is sufficient to estimate the correlation coefficient and associated 95% confidence interval with a lower bound of 0.14 and an upper bound of 0.45 (nQuery Advisor Version 7.0, Statsols, Cork, Ireland).

We used descriptive statistics to summarize demographic and clinical information on the participants. The differences between boys and girls were tested with Mann–Whitney *U* test for age, injury severity, worst pain, length of hospitalization, and severity of PTSS (total score CRIES) at 3 months, and with Fisher’s exact test for the presence of an extremity fracture. In addition, we calculated effect sizes using Cohen’s *d*. We examined whether variables followed a normal distribution by visually inspecting histograms. As the pain and CRIES scores clearly did not follow normal distributions, we used Spearman’s rho correlation coefficient to present the associations between variables. The following variables were included in the analyses: gender (as a fixed variable before the accident), the presence of an extremity fracture, the injury severity, the worst experienced pain, the length of hospitalization, and the total score of self-reported PTSS at 3 months (variables ordered in time from the moment of the accident). Correlation coefficients can be interpreted as .0 to (–).3 = *negligible*; (–).3 to (–).5 = *low*; (–).5 to (–).7 = *moderate*; (–).7 to (–).9 = *high*; (–).9 to –(1) = *very high* (Hinkle, Wiersma, & Jurs, 2003). A *p* value of less than .05 was considered statistically significant. Analyses were performed using SPSS 24 (IBM Statistical Product and Service Solutions, Chicago, IL).

## Results

### Sample Characteristics

Table 1 summarizes the sample characteristics for the total group of 135 children included in this study, and for boys and girls separately, and shows the differences and effect sizes between both groups on all variables. We found no significant differences between boys and girls, except on the severity of posttraumatic stress: girls had a higher CRIES score than boys. In total 94 children (70%) were hospitalized. ISS’s were available for 134 children. Injury severity was classified as zero for 31 children (23%), minor for 58 children (43%), moderate for 28 children (21%), serious for 12 children (9%), and severe for 5 children (4%). The number of days between the accident and the first assessment was not associated with the amount of pain (*r*_*s*_ = .04; *p* = .62) nor with the severity of PTSS (*r*_*s*_ = .04; *p* = .68).

### Acute Pain

When asked to rate the worst pain since the accident, the majority of the children reported moderate (*n* = 67, 50%) or severe (*n* = 58, 43%) pain, while ten children (7%) reported no to mild pain, including one child reporting no pain. The mean and median pain values are shown in Table 1. Severe pain was reported across all categories of injury severity. Ten children specifically reported medical procedures, like insertion of peripheral venous cannula or urethral catheterization, as very painful or most painful ever. The worst pain children reported referred to pain shortly after the accident while in the ambulance, during treatment in the trauma room, or during hospitalization.

### Posttraumatic Stress

On the self-report measure CRIES, the scores of 20 children (15%) were above the cut-off point (≥ 30), indicating serious PTSS. Additional information on the CRIES scores is reported in Table 1.

### Acute Pain and Posttraumatic Stress

The findings on the association between acute pain, child characteristics, and posttraumatic stress are summarized in Table 2. In the total group, the continuous pain score had a positive correlation (*r*_*s*_ = .28; *p* = .001) with the total score on the self-report measure CRIES. In girls, we found no significant association between acute pain and the total score on the CRIES (*r*_*s*_ = .23; *p* = .09). In boys, however, acute pain was significantly associated with the total score on the CRIES (*r*_*s*_ = .27; *p* = .02). After splitting the sample into pain categories, we found no significant association between continuous pain scores and the severity of posttraumatic stress for children with “no or mild pain” (*r*_*s*_ = .14; *p* = .71) or “moderate pain” (*r*_*s*_ = .13; *p* = .30). However, for children with “severe pain” this association was statistically significant (*r*_*s*_ = .32; *p* = .02). Separate examination of data for girls (*r*_*s*_ = .22; *p* = .28) and boys (*r*_*s*_ = .33; *p* = .07) revealed no statistically significant association between severe pain and posttraumatic stress, although these results are based on small subgroups.

**Table 2:** Correlations between acute pain, child characteristics, and posttraumatic stress

Total group (*n* = 135)	1	2	3	4	5
1. Pain score	–				
2. Extremity fracture	.17	–			
3. Injury severity	− .14	.50***	–		
4. Days hospitalized	.02	.49***	.83***	–	
5. Total score CRIES	.28**	.01	− .08	− .09	–

Girls (*n* = 56)	1	2	3	4	5
1. Pain score	–				
2. Extremity fracture	.04	–			
3. Injury severity	− .34*	.45**	–		
4. Days hospitalized	− .09	.44**	.78***	–	
5. Total score CRIES	.23	.11	− .11	− .02	–

Boys (n = 79)	1	2	3	4	5
1. Pain score	–				
2. Extremity fracture	.29*	–			
3. Injury severity	.04	.52***	–		
4. Days hospitalized	.15	.51***	.83***	–	
5. Total score CRIES	.27*	.00	− .03	− .09	–

Values are Spearman’s rho correlation coefficients (*r*_*s*_). *CRIES* Children’s Revised Impact of Event Scale

*p < .05

**p < .01

***p < .001

None of the other factors were associated with the total score of the CRIES, but two factors were associated with pain. In girls, injury severity was negatively associated with pain (*r*_*s*_ = − .34; *p* = .01); the more severe the injury was, the less pain the girls reported. In boys the presence of an extremity fracture was positively associated with pain (*r*_s_ = .29; *p* = .01); an extremity fracture was thus associated with more pain in boys but not in girls. Injury severity and the presence of an extremity fracture were associated factors, in the total group as well as in boys and girls separately.

## Discussion

In this study, we found an association between acute pain after accidental injury and PTSS 3 months later. The findings of our study confirm the findings of other studies, in that pain after accidental injury contributes to or is a risk factor for later PTSD or PTSS in children and adolescents (Hildenbrand et al., 2016; Saxe et al., 2005). However, the difference in outcome related to gender had not been specified previously. Furthermore, the association between acute pain and severity of posttraumatic stress was strongest in the group of children that reported severe pain. This result supports the finding of Hildenbrand et al. (2016) that the most severe pain predicted subsequent PTSS. In the group of children with severe pain, pain may account for around 10% of the variance in the severity of the PTSS after 3 months.

In our study, girls reported more severe PTSS than boys. This finding is consistent with a previous study which found that girls have a greater risk for PTSD then boys (Alisic et al., 2014). Injury severity and the presence of an extremity fracture were moderately associated and influenced the amount of acute pain. In boys, we found an association between the presence of an extremity fracture and pain. A possible explanation is that an extremity fracture causes more pain than other injuries. This is in line with the findings of Baxt et al. (2004) in which extremity fracture was associated with greater “worst pain” ratings. Pain management may not always fit the need for pain medication that accompanies the presence of an extremity fracture, at least not immediately. Except for the gender difference that emerged from our study, our results are in line with previous findings (Winston et al., 2003), i.e., that the presence of one or more extremity fractures is considered to be a risk factor for persistent posttraumatic stress. Although previous research has shown that injury severity is not a predictor of PTSD (Brosbe et al., 2011), we found a negative association between injury severity and pain. A possible explanation might be that more severely injured children are likely to receive more adequate pain medication. The negative association was only found in girls.

Research on gender differences in pain suggests a difference between genders in their response to pain. Gender has been reported as a critical factor in the perception of pain; males and females experience pain differently (Paller, Campbell, Edwards, & Dobs, 2009). In that study, increased pain sensitivity and risk for clinical pain were more common in women. The specific basis for the differences between genders is still unknown, but research suggests that multiple biological and psychosocial processes are involved (Bartley & Fillingim, 2013). Furthermore, differences between genders might be related to a difference in communication and openness about the degree of pain. This is in line with the suggestion in Paller et al. (2009) that boys and girls are reinforced differently for their expression of pain-related experiences. Another possible explanation is that medical staff evaluates the degree of pain differently in boys and girls. Although girls generally have a greater risk for PTSD than boys (Alisic et al., 2014; Stallard et al., 2004; Winston et al., 2003), our results suggest that the risk for PTSD in injured children might be influenced by injury severity, pain, and pain management.

In the context of our findings, the subjectivity of reported pain should be addressed. Besides the injury itself, psychological mechanisms, like fear and loss of control, play a role in mediating the pain. Many people report pain for psychological reasons (International Association for the Study of Pain [IASP], 2017). There is no way to distinguish the subjective reporting of pain from pain that is due to tissue damage. According to the IASP, if people regard their experience as pain, it should be accepted as pain. This definition avoids tying pain to the stimulus. This clearly indicates the importance of pain measurement and subsequent pain medication according to the patient’s report as stated in pain protocols. In a review of the availability and content of acute pain protocols in emergency departments in the Netherlands, the authors emphasized the importance of adequate acute pain control, not only from the perspective of good patient care, but also due to adverse physical effects and the risk of developing chronic pain (Gaakeer, van Lieshout, & Bierens, 2010). The latter is strongly associated with chronic PTSD (Chossegros et al., 2011). From the responding Dutch emergency departments, 35% did not have a pain management protocol for children (Gaakeer et al., 2010), which heightens the risk of misjudgment and undertreatment. Several studies lend further support to the relationship between pain and later PTSD development by describing how aggressive pharmacological pain management at the time of initial hospitalization can reduce the likelihood of PTSD development (Gold et al., 2008).

In addition to medication, the use of psychological strategies (e.g., distraction) by the medical staff can be of great help in reducing the subjective experience of pain, whether or not mediated by relief of anxiety. They can be applied dependent on the situation and the child’s characteristics and preferences (Koller & Goldman, 2012; Langeland & Olff, 2008). Furthermore, Trauma Informed Care (TIC) offers a multidisciplinary approach to reduce the risk for persisting posttraumatic stress and PTSD following injury (Marsac et al., 2016; Weiss et al., 2017). TIC is characterized by realizing the effect of trauma, recognizing how trauma can affect those involved, bringing trauma-related knowledge into practice and preventing further negative reactions (Marsac et al., 2016). Implementing TIC can increase medical staff awareness of stressors following injury and can provide them with strategies that can help minimize the adverse effect of these stressors.

## Strengths and Limitations

Due to the nature of our study and the acute situation after an accident, a retrospective pain rating was used, which increases the chance of unreliable ratings. Some of the children may have reported less reliably on the worst experienced pain, due to a period of unconsciousness or amnesia. Since we only used a single pain scale, we could not perform sensitivity analyses using another instrument. Moreover, pain medication could have had a confounding effect on the outcome but we were unable to account for this possible effect. The administration of pain medication was reported in the medical records, but children reported the worst experienced pain retrospectively. They did not report the exact moment in time that they experienced this pain. Therefore, we were unable to relate pain to information on pain medication. Additionally, we did not assess pain over time, although this could have provided more insight into the relationship between pain and the other variables. Furthermore, only the presence or absence of an extremity fracture was specifically registered as part of the STEPP study. We therefore did not include other types of injury classifications as an independent variable in the current study. Baseline acute stress may have contributed to the report of pain at baseline and to posttraumatic stress at 3 months but this was not assessed in our study.

Ideally, we would have examined the relationship between acute pain and a diagnosis of PTSD or significant PTSS. In this case, logistic regression analysis would have been appropriate. We would then have examined differences between the relationships between these variables for boys and girls using interaction effects. However, the number of children with significant PTSS (*n* = 20) or PTSD (van Meijel et al., 2015) in our sample was too low to perform logistic regression analysis, including a correction for gender and other potentially relevant factors (Peduzzi, Concato, Kemper, Holford, & Feinstein, 1996). Because of skewness of the data (many children without symptoms and few children with low pain scores), a transformation of the data offered no solution. The use of bivariate correlation precludes corrections for multiple factors. However, the use of Spearman’s rho correlation coefficients was the best alternative to obtain a reliable answer to the research question, certainly because there is a definite ordering of most of the variables in time. Although correlation coefficients should be interpreted as associations, the succession in time of the variables creates possibilities for additional interpretations. However, only an association, and not causation, can be inferred from cross-sectional data (Sedgwick, 2014). An advantage of the method we used was that pain and severity of posttraumatic stress were reported directly by the children and therefore were not biased by the interpretation of parents or professionals.

## Conclusions and Clinical Implications

This study contributes to the knowledge of factors related to the risk of posttraumatic stress following accidental injury, specifically regarding acute pain. The experience of pain may be subjective, but severe acute pain is associated with the severity of later PTSS. We therefore recommend timely measurement and management of pain according to acute pain protocols in all phases and disciplines after accidental injury. Further research is needed to investigate the role of gender, to clarify the interaction between pain, injury, and injury severity, and to examine the usefulness of acute pain in screening tools.

## References

[CR1] Alisic E, Zalta AK, van Wesel F, Larsen SE, Hafstad GS, Hassanpour K, Smid GE (2014). Rates of post-traumatic stress disorder in trauma-exposed children and adolescents: Meta-analysis. The British Journal of Psychiatry.

[CR2] American College of Surgeons. (2012). Advanced trauma life support (9th ed., Fig. 1–2, step 3). Retrieved from https://www.44c.in.ua/files/book11.pdf.

[CR3] APA (2000). Diagnostic and statistical manual of mental disorders.

[CR4] APA (2013). Diagnostic and statistical manual of mental disorders.

[CR5] Asmundson GJ, Coons MJ, Taylor S, Katz J (2002). PTSD and the experience of pain: Research and clinical implications of shared vulnerability and mutual maintenance models. Canadian Journal of Psychiatry. Revue Canadienne De Psychiatrie.

[CR40] Baas E, Kramer RP (2008). Protocol pijnmeetinstrumenten (protocol painmeasurement).

[CR6] Baker SP, O’Neill B, Haddon W, Long WB (1974). The injury severity score: A method for describing patients with multiple injuries and evaluating emergency care. The Journal of Trauma.

[CR7] Bartley EJ, Fillingim RB (2013). Sex differences in pain: A brief review of clinical and experimental findings. British Journal of Anaesthesia.

[CR8] Baxt C, Kassam-Adams N, Nance ML, Vivarelli-O’neill C, Winston FK (2004). Assessment of pain after injury in the pediatric patient: Child and parent perceptions. Journal of Pediatric Surgery.

[CR9] Brosbe MS, Hoefling K, Faust J (2011). Predicting posttraumatic stress following pediatric injury: A systematic review. Journal of Pediatric Psychology.

[CR10] Children and War Foundation. (1998). Instruction and English version of the Children’s Revised Impact of Event Scale (CRIES-13). Retrieved from http://www.childrenandwar.org/measures/children%E2%80%99s-revised-impact-of-event-scale-8-%E2%80%93-cries-8/ies13/.

[CR11] Chossegros L, Hours M, Charnay P, Bernard M, Fort E, Boisson D, Laumon B (2011). Predictive factors of chronic post-traumatic stress disorder 6 months after a road traffic accident. Accident; Analysis and Prevention.

[CR12] Dalgleish T, Meiser-Stedman R, Kassam-Adams N, Ehlers A, Winston F, Smith P, Yule W (2008). Predictive validity of acute stress disorder in children and adolescents. The British Journal of Psychiatry.

[CR13] Gaakeer MI, van Lieshout JM, Bierens JJ (2010). Pain management in emergency departments: A review of present protocols in The Netherlands. European Journal of Emergency Medicine.

[CR14] Gold JI, Kant AJ, Kim SH (2008). The impact of unintentional pediatric trauma: A review of pain, acute stress, and posttraumatic stress. Journal of Pediatric Nursing.

[CR15] Hildenbrand AK, Marsac ML, Daly BP, Chute D, Kassam-Adams N (2016). Acute pain and posttraumatic stress after pediatric injury. Journal of Pediatric Psychology.

[CR16] Hinkle DE, Wiersma W, Jurs SG (2003). Applied statistics for the behavioral sciences.

[CR17] International Association for the Study of Pain [IASP]. (2017). IASP taxonomy. Retrieved from https://www.iasp-pain.org/Education/Content.aspx?ItemNumber=1698#Pain.

[CR18] Kahana SY, Feeny NC, Youngstrom EA, Drotar D (2006). Posttraumatic stress in youth experiencing illnesses and injuries: An exploratory meta-analysis. Traumatology.

[CR19] Keene DD, Rea WE, Aldington D (2011). Acute pain management in trauma. Trauma.

[CR20] Koller D, Goldman RD (2012). Distraction techniques for children undergoing procedures: A critical review of pediatric research. Journal of Pediatric Nursing.

[CR21] Landolt MA, Vollrath M, Timm K, Gnehm HE, Sennhauser FH (2005). Predicting posttraumatic stress symptoms in children after road traffic accidents. Journal of the American Academy of Child and Adolescent Psychiatry.

[CR22] Langeland W, Olff M (2008). Psychobiology of posttraumatic stress disorder in pediatric injury patients: A review of the literature. Neuroscience and Biobehavioral Reviews.

[CR23] Marsac ML, Kassam-Adams N, Marsac ML, Kassam-Adams N, Hildenbrand AK, Nicholls E, Fein J (2016). Implementing a Trauma-Informed Approach in pediatric health care networks. JAMA Pediatrics.

[CR24] McLean SA, Clauw DJ, Abelson JL, Liberzon I (2005). The development of persistent pain and psychological morbidity after motor vehicle collision: Integrating the potential role of stress response systems into a biopsychosocial model. Psychosomatic Medicine.

[CR25] Melby V, McBride C, McAfee A (2011). Acute pain relief in children: Use of rating scales and analgesia. Emergency Nurse.

[CR26] Morley S (1993). Vivid memory for ‘everyday’ pains. Pain.

[CR27] Norman SB, Stein MB, Dimsdale JE, Hoyt DB (2008). Pain in the aftermath of trauma is a risk factor for post-traumatic stress disorder. Psychological Medicine.

[CR28] Olff, M. (2005). Instruction and Dutch version of the Children’s Revised Impact of Event Scale (CRIES-13). Retrieved from http://www.childrenandwar.org/measures/children%E2%80%99s-revised-impact-of-event-scale-8-%E2%80%93-cries-8/ies13/.

[CR29] Paller CJ, Campbell CM, Edwards RR, Dobs AS (2009). Sex-based differences in pain perception and treatment. Pain Medicine.

[CR30] Peduzzi P, Concato J, Kemper E, Holford TR, Feinstein AR (1996). A simulation study of the number of events per variable in logistic regression analysis. Journal of Clinical Epidemiology.

[CR31] Price J, Kassam-Adams N, Alderfer MA, Christofferson J, Kazak AE (2016). Systematic review: A reevaluation and update of the integrative (trajectory) model of pediatric medical stress. Journal of Pediatric Psychology.

[CR32] Saxe GN, Miller A, Bartholomew D, Hall E, Lopez C, Kaplow J, Moulton S (2005). Incidence of and risk factors for acute stress disorder in children with injuries. The Journal of Trauma.

[CR33] Sedgwick P (2014). Cross sectional studies: Advantages and disadvantages. British Medical Journal.

[CR34] Stallard P, Salter E, Velleman R (2004). Posttraumatic stress disorder following road traffic accidents—A second prospective study. European Child & Adolescent Psychiatry.

[CR35] Stoddard FJ, Saxe G, Ronfeldt H, Drake JE, Burns J, Edgren C, Sheridan R (2006). Acute stress symptoms in young children with burns. Journal of the American Academy of Child and Adolescent Psychiatry.

[CR36] van Meijel EP, Gigengack MR, Verlinden E, Opmeer BC, Heij HA, Goslings JC, Lindauer RJ (2015). Predicting posttraumatic stress disorder in children and parents following accidental child injury: Evaluation of the Screening Tool for Early Predictors of Posttraumatic Stress Disorder (STEPP). BMC Psychiatry.

[CR37] Verlinden E, van Meijel EP, Opmeer BC, Beer R, de Roos C, Bicanic IA, Lindauer RJ (2014). Characteristics of the children’s revised impact of event scale in a clinically referred dutch sample. Journal of Traumatic Stress.

[CR38] Weiss D, Kassam-Adams N, Murray C, Kohser KL, Fein JA, Winston FK, Marsac ML (2017). Application of a framework to implement trauma-informed care throughout a pediatric health care network. The Journal of Continuing Education in the Health Professions.

[CR39] Winston FK, Kassam-Adams N, Garcia-Espana F, Ittenbach R, Cnaan A (2003). Screening for risk of persistent posttraumatic stress in injured children and their parents. JAMA.

